# Microsporidial keratitis retrospectively diagnosed by ultrastructural study of formalin-fixed paraffin-embedded corneal tissue: a case report

**DOI:** 10.1186/s12941-019-0316-y

**Published:** 2019-06-10

**Authors:** Satoru Ueno, Hiroshi Eguchi, Fumika Hotta, Masahiko Fukuda, Masatomo Kimura, Kenji Yagita, Takashi Suzuki, Shunji Kusaka

**Affiliations:** 1 Department of Ophthalmology, Kindai University Faculty of Medicine, 377-2, Ohnohigashi, Osakasayama, Osaka, 589-8511 Japan; 2 Department of Pathology, Kindai University Faculty of Medicine, 377-2, Ohnohigashi, Osakasayama, Osaka, 589-8511 Japan; 3 Department of Parasitology, National Institute of Infectious Diseases, 1-23-1, Toyama, Shinjuku, Tokyo, 162-8640 Japan; 4 Department of Ophthalmology, Toho University Medical Center, 6-11-1, Ohmorinishi, Ota, Tokyo, 143-8541 Japan

**Keywords:** Microsporidial keratitis, Descemet stripping automated endothelial keratoplasty, Formalin-fixed paraffin-embedded, Transmission electron microscopy

## Abstract

**Background:**

The utility of formalin-fixed paraffin-embedded (FFPE) corneal tissue specimens for retrospective diagnosis of microsporidial keratitis was evaluated by transmission electron microscopy (TEM) analysis and the possible second case of microsporidial keratitis after Descemet stripping automated endothelial keratoplasty (DSAEK) was described.

**Case presentation:**

A 68-year-old man presented with multiple crystalline opacities in the corneal stroma that progressed extremely slowly after DSAEK. Fungiflora Y staining of corneal scrapings from the affected regions revealed an oval microorganism. Topical voriconazole administration was ineffective and penetrating keratoplasty was performed. Histological and molecular analyses were carried out on the excised cornea. Ziehl–Neelsen staining revealed an acid-fast, oval organism that was visible by ultraviolet illumination after Fungiflora Y and Uvitex 2B staining, whereas periodic acid-Schiff and Grocott’s staining did not yield any significant findings. Microsporidium was detected by TEM of FFPE tissue. *Nosema* or *Vittaforma* sp. was suspected as the causative microorganism by PCR of FFPE tissue and by the fact that those species are known to cause eye infection. The corneal graft has maintained transparency at 1 year and half postoperatively.

**Conclusions:**

This is the first known case of microsporidial keratitis diagnosed retrospectively by molecular and ultrastructural study of FFPE tissue, and the possible second case of microsporidial keratitis after DSAEK. Microsporidial keratitis should be considered when corneal opacity refractory to conventionally known therapy would occur after DSAEK. Our findings suggest that more microsporidial keratitis cases than have been reported to date can be identified by TEM or PCR examination of FFPE corneal specimens.

## Background

Microsporidia constitute a group of obligate intracellular organisms encompassing several genera. These small, oval, eukaryotic intracellular spore-forming protozoan parasites are widely distributed in vertebrates and invertebrates. Microsporidial keratitis was first reported in 1973 [1] and was initially thought to be associated with immunocompromised hosts [2–4]. Awareness of microsporidial keratitis has increased in recent years [5] and a few cases have been reported, especially in tropical and semitropical countries. However, there is only one documented case of microsporidial keratoconjunctivitis after Descemet striping automated endothelial keratoplasty (DSAEK) [6]. Since the number of microsporidial keratitis reports from countries with extratropical, subarctic, and polar climates is so small, the exact incidence in these regions is unknown.

Diagnosing microsporidial keratitis is challenging for ophthalmologists for two reasons. Firstly, the organism cannot be easily detected by routine examination in clinical settings. Most clinicians and laboratory technicians are unfamiliar with the optimal conditions for the growth of microsporidia, which are fastidious. For a rigorous diagnosis, corneal specimens must be subjected to acid-fast staining, which is not always feasible in practice. Secondly, clinical manifestations of microsporidial keratitis include a nummular pattern [7–9], epithelial opacity, diffuse epithelial keratopathy, punctate epithelial erosion, or stromal keratitis, which can result in misdiagnosis as fungal or bacterial keratitis or sterile corneal infiltration. It is, therefore, possible that there have been more microsporidial keratitis cases than have been reported and/or diagnosed to date by conventional methods. Given that a case of microsporidial keratitis masquerading as graft rejection after DSAEK has been reported [6], it is necessary for corneal surgeons to always consider microsporidial keratitis in cases of any type of corneal opacity after DSAEK. If microsporidia can be identified in formalin-fixed paraffin-embedded (FFPE) corneal specimens that have already been diagnosed as another disease, it may be possible to retrospectively determine with accuracy the actual number of microsporidial keratitis cases, which would be useful for clarifying the clinical characteristics of this condition for ophthalmologists.

In this case report, the utility of ultrastructural analysis of FFPE corneal specimens for retrospective diagnosis of microsporidial keratitis was evaluated for the first time and the possible second documented case of microsporidial keratitis after DSAEK was described.

## Case presentation

**Fig. 1:**
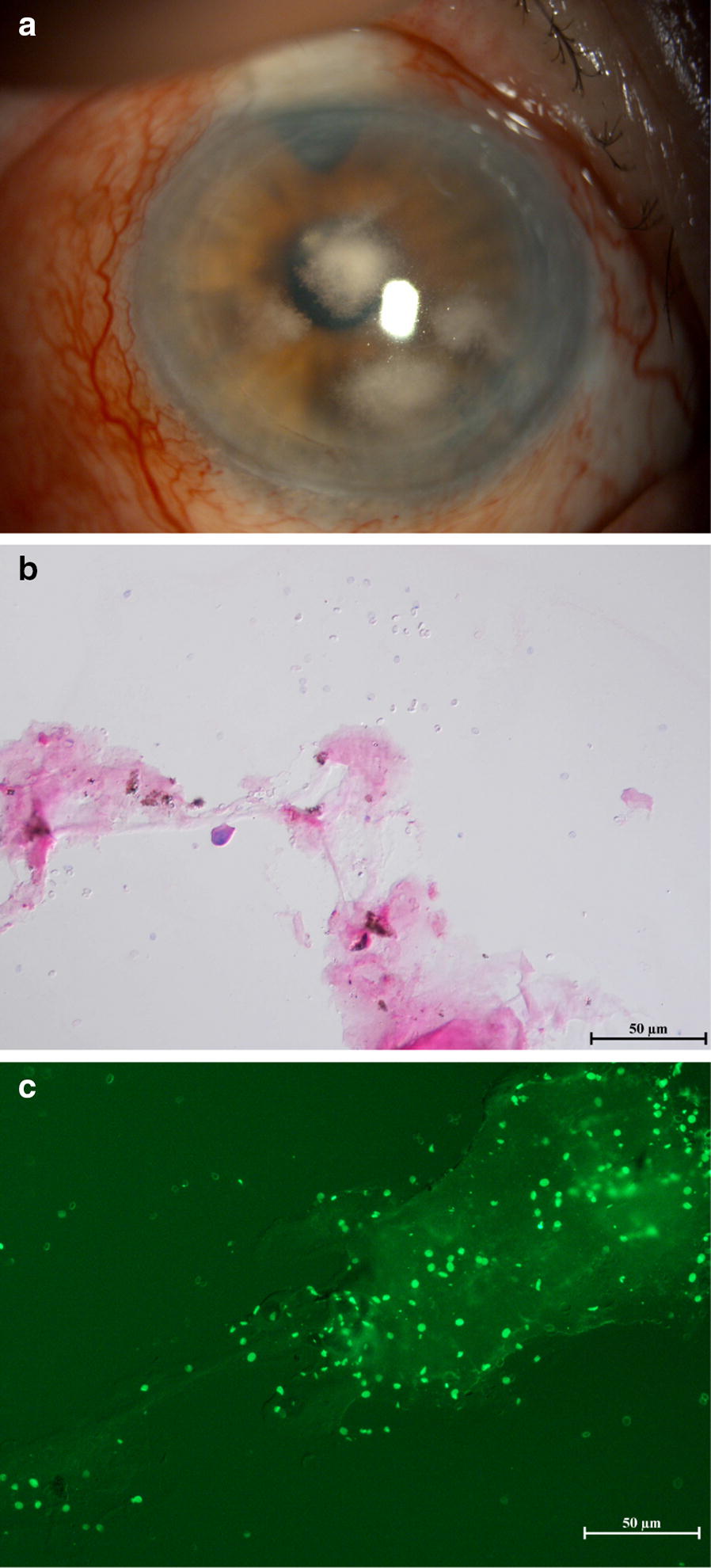
**a** Image of anterior segment before penetrating keratoplasty. Multiple corneal opacities showing crystalline keratopathy were observed. **b** Gram staining of the scraped cornea (×400 magnification). Although oval microorganisms were detected, they were not stained. **c** Fungiflora Y staining of the scraped cornea (×400 magnification). Oval microorganisms were visible under ultraviolet illumination

A 68-year-old man was referred to Kindai University in 2004 with bilateral uveitis of unknown cause. The right eye had lost vision due to suspected *Candida* keratitis after penetrating keratoplasty, which was performed in 2007. A mild anterior chamber inflammation and keratic precipitates with small corneal oedema, followed by refractory secondary glaucoma, caused bullous keratopathy in the left eye that necessitated DSAEK in 2011. The clinical findings observed during these periods, such as unilateral high intraocular pressure and corneal oedema with keratic precipitates, were suggestive of cytomegalovirus (CMV) corneal endotheliitis. A diagnosis of CMV corneal endotheliitis was made based on detection of CMV DNA in the aqueous humour after DSAEK. Corneal grafting failed even with administration of 0.5% ganciclovir eye drop six times with 0.1% fluorometholone eye drop four times daily for more than a year. After the second DSAEK in 2013, 1.0% voriconazole, 0.5% ganciclovir, and 0.1% betamethasone phosphate eye drops continued to be administered four times daily for 2 years. In 2015, the patient presented with small crystalline opacities in the centre of the cornea that progressed extremely slowly and had multiplied by 2017 (Fig. 1a). The patient complained visual disturbance without any eye pain or foreign body sensation when the corneal opacity covered the visual axis, although he did not exhibit any subjective symptoms when the keratitis occurred for the first time. Gram staining of the scraped cornea revealed an unstained small oval microorganism (Fig. 1b) that was only visible by Fungiflora Y staining (Fig. 1c). Given the past episode of vision loss of the other eye due to suspected *Candida* keratitis, we administered two doses of voriconazole by intrastromal injection. Since the treatment was ineffective, penetrating keratoplasty was performed. The excised corneal tissue was fixed with formalin, embedded in paraffin, and processed for histological analysis.

**Fig. 2:**
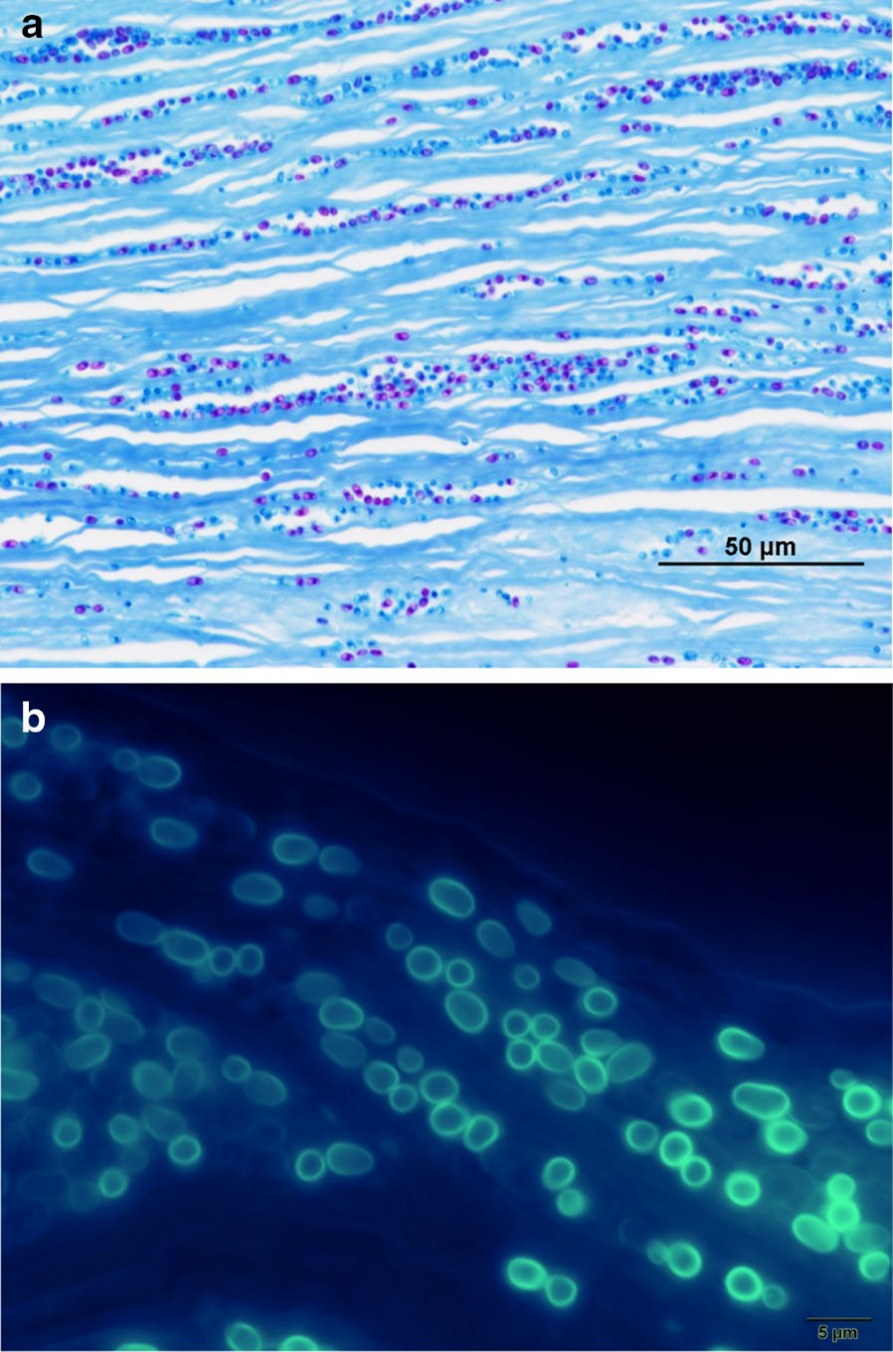
**a** Ziehl–Neelsen staining of the excised cornea. Oval microorganisms stained with a reddish purple colour were observed. **b** Uvitex 2B staining of the excised cornea. Oval microorganisms showing blue fluorescence were visible

**Fig. 3:**
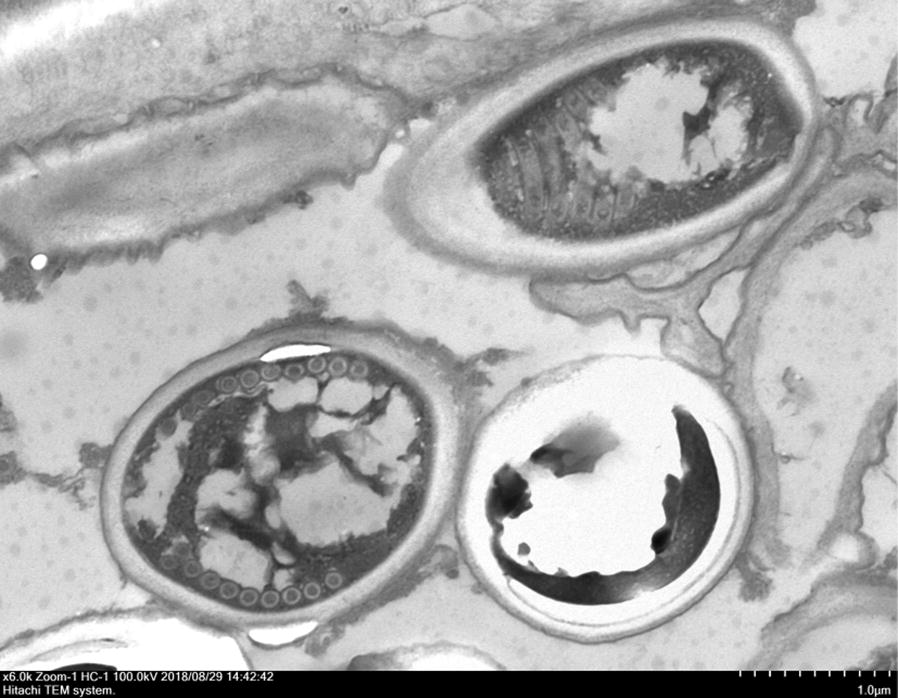
Transmission electron micrograph of paraffin-embedded excised corneal tissue. A mature spore with approximately 10 polar tube coils can be seen at high magnification

Histologically, numerous oval organisms, 1.3–2.6 μm in diameter, were found throughout the corneal stroma. The organisms could be identified by haematoxylin and eosin staining and Ziehl–Neelsen staining, and fluoresced under ultraviolet illumination by Fungiflora Y and Uvitex 2B staining, but were unstained with periodic acid-Schiff reaction and Grocott’s staining (Fig. 2a, b). For TEM observation, ultrathin sections were prepared from the targeted area of paraffin sections after osmification and embedding in Epon blocks by the inverted beam capsule method [10]. They were observed with an HT 7700 microscope (Hitachi High-Technologies, Tokyo, Japan). A polar tube with multiple loose coils—which is consistent with the morphology of microsporidia—was detected in the spore-like elements of the microorganisms by TEM (Fig. 3). The corneal graft remains transparent and no clinical findings suggestive of recurrence of microsporidial keratitis nor graft rejection is found with administration of 0.1% betamethasone phosphate eye drop four times daily at 1 year and half postoperatively.

## Discussion and conclusions

Most clinicians assume that FFPE tissue specimens are only useful for pathological examinations by light and/or fluorescence microscopy. However, there are several reports of FFPE tissues being used for molecular or electron microscopic analyses in the field of diagnostic pathology [10–12] or for proteomic investigation [13]. If archival TEM and/or molecular analyses of FFPE tissues were more widely adopted by ophthalmologists, more corneal and/or external diseases of unknown cause that were treated by surgery could be accurately diagnosed. Fastidious organisms in difficult-to-diagnose infectious diseases can also be identified by TEM or PCR, which would provide an accurate survey of epidemiological conditions. In usual clinical course, microsporidial keratitis is diagnosed in a prospective manner for clinical need [1–9, 14–17]. Therefore, ophthalmologists have to optimize the culture condition for microsporidia when they encounter the keratitis. Otherwise, they have to choose the optimal pathological methods after excision of the cornea. If the ophthalmologists were unfamiliar with microsporidia and had no idea of a retrospective diagnosing method, microsporidial keratitis would not be noticed. The present case report, to the best of our knowledge, is the first example in ophthalmology of TEM being applied to an FFPE sample in a retrospective manner. Given that microsporidia are easily overlooked or confused with small yeast [18], we should have also re-examined an FFPE corneal sample of the other eye, but this was not possible because it had been discarded.

With respect to molecular diagnosis, we tried to make accurate diagnosis by extracting microsporidial DNA from the FFPE sample using commercially available product according to the manufacturer’s instructions; this served as a template for polymerase chain reaction (PCR) amplification, which was performed following a protocol that can identify *Enterocytozoon* and *Encephalitozoon* species of microsporidia. A PCR product was obtained using pan-microsporidial primers, although none were obtained using primers specific for *Encephalitozoon* or *Enterocytozoon* species. Eventually, precise species identification was not achieved.

*Nosema* and *Vittaforma* sp. are known to cause eye infection in healthy immunocompetent patients [14, 15, 19]. *Encephalitozoon* or *Enterocytozoon* has been reported as a pathogenic genus in several organs in cases of immunodeficiency [2, 3, 16, 20]. Based on the known medical history, our patient was a healthy elderly man, which is consistent with the above-mentioned epidemiology if the causative strain of this patient was *Nosema* sp. or *Vittaforma* sp. The reason for differences in the affinity of microsporidial species according to the human immune status is yet to be determined. We speculate that *Nosema* or *Vittaforma* in this patient originated from the natural environment since he lives in a rural area and the corneal epithelial defect was observed at the onset of keratitis.

Topical voriconazole monotherapy has been used to treat microsporidial keratitis [17]. However, in this case, voriconazole was not effective even when it was administered as an eye drop for several years and twice by intrastromal injection. Not only topical voriconazole, but also topical steroids were administered over a long period. Non-specific immunodeficiency on the ocular surface caused by the latter may promote chronic microsporidial infection in the cornea. Therefore, we recommend that patients who have undergone keratoplasty with long-term administration of topical steroids should be carefully examined for potential microsporidial keratitis, even if they are immunocompetent. Albendazole and fumagillin [21] should be considered as treatment options for recurrent microsporidial keratitis.

Two limitations in the interpretation of TEM images and PCR results are included in this case report. It is not guaranteed that TEM of FFPE tissue of microsporidial infections can detect the characteristic structure of microsporidia in all cases. In the current case, the inner structure of the spore was broken, although the cross section of the polar tube was confirmed. Whether high quality TEM images can be obtained or not may depend on the technique of ultra-thin sectioning. Presumably, the reason why species identification by PCR was not achieved is because very low amount of microsporidial DNA was extracted from FFPE tissue. We think that the success rate of PCR species identification may depend on the amount of microsporidial spores in the FFPE tissue, which means the small-sized tissue, such as the cornea, is disadvantageous. Further case accumulation or basic research is needed for overcoming these limitations.

In conclusion, to the best of our knowledge, the first known case of microsporidial keratitis diagnosed retrospectively by molecular and ultrastructural study of FFPE tissue and the possible second one after DSAEK was reported. In the future, the precise clinical features of microsporidial keratitis can be identified by performing PCR and TEM analyses of FFPE corneal specimens excised for the purpose of investigating other corneal diseases. Ophthalmologists should be aware of the utility of FFPE tissue samples for retrospective diagnoses of diseases of unknown cause.

## Data Availability

The datasets for the current study are available from the corresponding author on reasonable request.

## Abbreviations

FFPE: formalin-fixed paraffin-embedded
TEM: transmission electron microscopy
DSAEK: Descemet stripping automated endothelial keratoplasty
CMV: cytomegalovirus
PCR: polymerase chain reaction
